# Effects of Plasma Treatment on the Surface and Photocatalytic Properties of Nanostructured SnO_2_–SiO_2_ Films

**DOI:** 10.3390/ma16145030

**Published:** 2023-07-16

**Authors:** Igor A. Pronin, Alexander P. Sigaev, Alexei S. Komolov, Evgeny V. Zhizhin, Andrey A. Karmanov, Nadezhda D. Yakushova, Vladimir M. Kyashkin, Konstantin N. Nishchev, Victor V. Sysoev, Sanket Goel, Khairunnisa Amreen, Ramya K, Ghenadii Korotcenkov

**Affiliations:** 1 Department of Nano- and Microelectronics, Penza State University, 440026 Penza, Russia; alexsigaev-94@yandex.ru (A.P.S.); starosta07km1@gmail.com (A.A.K.); yand93@mail.ru (N.D.Y.); 2 Resource Center “Physical Methods of Surface Investigation”, St. Petersburg State University, 199034 St. Petersburg, Russia; a.komolov@spbu.ru (A.S.K.); evgeniy.zhizhin@spbu.ru (E.V.Z.); 3 Institute of Physics and Chemistry, Ogarev Mordovia State University, 430005 Saransk, Russia; kyashkin@mail.ru (V.M.K.); nishchev@inbox.ru (K.N.N.); 4 Department of Physics, Yuri Gagarin State Technical University of Saratov, 410054 Saratov, Russia; 5 MEMS, Microfluidics and Nanoelectronics Lab, Birla Institute of Technology and Science, Hyderabad 500078, India; 6 Department of Physics and Engineering, Moldova State University, 2009 Chisinau, Moldova; ghkoro@yahoo.com

**Keywords:** plasma treatment, sol-gel, tin dioxide, X-ray photoelectron spectroscopy

## Abstract

In this work, we study the effects of treating nanostructured SnO_2_–SiO_2_ films derived by a sol-gel method with nitrogen and oxygen plasma. The structural and chemical properties of the films are closely investigated. To quantify surface site activity in the films following treatment, we employed a photocatalytic UV degradation test with brilliant green. Using X-ray photoelectron spectroscopy, it was found that treatment with oxygen plasma led to a high deviation in the stoichiometry of the SnO_2_ surface and even the appearance of a tin monoxide phase. These samples also exhibited a maximum photocatalytic activity. In contrast, treatment with nitrogen plasma did not lead to any noticeable changes in the material. However, increasing the power of the plasma source from 250 W to 500 W led to the appearance of an SnO fraction on the surface and a reduction in the photocatalytic activity. In general, all the types of plasma treatment tested led to amorphization in the SnO_2_–SiO_2_ samples.

## 1. Introduction

The predominant focus of current research is on the photocatalytic properties of materials based on wide-gap semiconductor oxides, including TiO_2_, ZnO, SnO_2_ [1,2,3], independent of their other functional properties related to adsorption, sensor capabilities, etc. Apparently, this circumstance is due to differences in functional media: the photocatalytic reactions are carried out mainly in an aqueous medium [4,5], while the natural environment for the operation of the devices based on semiconductor oxides, except for special cases, is not aqueous.

Among the wide variety of semiconductor oxides, tin dioxide SnO_2_ occupies a special place. This material is distinguished by its non-toxicity, chemical inertness, stability of structural and physical properties, and economic efficiency. It is widely used in manufacturing the gas-sensitive layers used to detect gas molecules in the atmosphere [6], and in the electrodes in batteries [7], and so on. The major disadvantage to employing SnO_2_ as a material for photocatalysis is its high rate of recombination of electron–hole pairs. Since the discovery of the photocatalytic properties of tin dioxide by Wrighton in 1976 [8], a great number of technological approaches to improve the quantum efficiency of photocatalytic processes in SnO_2_ have been developed. These approaches can be divided into three broad groups: development of semiconductor composites, control of morphology, and management of structural energy design [9].

However, until now, the prospects for using tin dioxide as an effective photocatalyst remain doubtful, although this does not minimize its importance for applications in some other areas like sensorics [10], electrochemistry [11], biomedical applications [12], etc. In these areas, manufacturing devices based on SnO_2_ often requires a plasma treatment, which significantly contributes to a modification of its adsorption, sensor and several other properties that are largely lost [13,14].

Herein, we study the effect of plasma treatment of tin dioxide films on their surface and photocatalytic properties, which are selected for analysis due to their close relationship with the adsorption and sensor functionalities. While such processing is frequently employed as a manufacturing operation to produce semiconductor devices [15,16,17,18], it has also been found that oxygen plasma treatment improves the performance of metal oxide gas sensors [13,19,20]. The analysis of literature sources also shows that plasma treatment leads to a significant improvement in the photocatalytic properties of semiconductor oxides. For example, the researchers [21] demonstrated a 2.8-fold increase in the rate constant of the decomposition of methyl orange on titanium dioxide samples treated with nitrogen plasma in visible radiation compared with the initial rates. The article [22] showed a more than eight-fold increase in the decomposition rate constant of methyl orange on zinc oxide samples treated with dielectric barrier discharge plasma in various modes. The work [23] demonstrated the appearance of photocatalytic activity in visible light in TiO_2_ samples treated with hydrogen plasma. The researchers [24] noted that treating WO_3_ nanoparticles with radio frequency hydrogen plasma led to an improvement in the photocatalytic activity of the water photolysis process.

Therefore, it is important to know the effect of such treatments on the properties of metal oxides, which are promising for the noted applications. The material, of 0.85SnO_2_–0.15SiO_2_ composition, obtained by the sol-gel method is chosen for the object of study. Earlier, it was shown [15] that the indicated composition is close to the optimal for developing gas-sensor structures with efficient functional characteristics. In addition, this composition is selected as it forms films with the developed surface and hierarchical labyrinth structure, which ensures improved photocatalytic properties compared with a homogeneous or granular material [25,26]. Our previous studies have shown [27] that the addition of 15 at. % silicon dioxide to tin dioxide makes it possible to obtain structures of this kind by the sol-gel method. X-ray photoelectron spectroscopy is considered here as a major method for studying the surface. Photocatalytic activity is employed to evaluate the surface reactivity of the samples under study.

## 2. Materials and Methods

### 2.1. Synthesis of Samples

The thin films of 0.85SnO_2_–0.15SiO_2_ composition were derived in frames of sol-gel technology by the sol immersion method. The following precursors were used to prepare the initial sol: tin chloride pentahydrate SnCl_4_·5H_2_O (≥99.0%); tetraethoxysilane Si(OC_2_H_5_)_4_ (≥99.0%), isopropyl alcohol CH_3_CH(OH)CH_3_ (≥99.0%); and hydrochloric acid (37 wt. %). All the precursors were of analytical purity (Aldrich-Sigma). At the first stage, 4.4 g of SnCl_4_·5H_2_O was dissolved in 100 mL of isopropyl alcohol followed by the addition of 0.5 mL of Si(OC_2_H_5_)_4_ and 50 μL of HCl (37 wt. %). The resulting solution was then stirred constantly for 30 min. The output sol matured for 24 h at room temperature. After maturation, the sol was applied to glass substrates by immersion using the Xdip-MV1 automated coating system (Apex Instruments Co., Kolkata, India) with the following parameters: the rate of immersion in the sol was 10 mm/min; the holding time in the sol was 30 s; the rate of extraction from the sol was 10 mm/min; and the drying time in the air was 30 s. After filming, the samples were subjected to pre-drying at 65 °C for 25 min and final thermal annealing at 600 °C for 60 min in the normal air atmosphere. These samples are designated in this work as PL0.

The resulting basic samples PL0 were processed in oxygen and nitrogen plasmas using the SI 500 inductively coupled plasma etcher (SENTECH Instruments GmbH, Berlin, Germany). The treatment modes and the corresponding parameters are listed in Table 1. The plasma treatment was applied in a chamber at the operating pressure of 0.2 Pa under the gas flowrate of 25 SCCM. The temperature of the working table in the etcher chamber was 10 °C, and the etching time was 30 s. For the PL1 and PL2 modes, nitrogen and oxygen plasmas with the source power of 250 W were used, which ensured the sample etching depth comparable to half the film thickness. The PL3 mode consisted of nitrogen plasma with a source power of 500 W. The same power of the oxygen plasma source led to destruction of the film structure and its etching to the substrate.

Therefore, it was used in this work. The PL4 mode consisted of nitrogen plasma with the power of 250 W with the additional 10 W high-frequency source to be connected. For these reasons, the PL1 and PL2 modes are characterized as “soft”, and the modes PL3 and PL4 are characterized as “hard”.

### 2.2. Electron Microscopy

The surface structure of the samples was studied using the scanning electron microscope VEGA 3 SBH (TESCAN, Brno, Czech Republic) with the reflection electron detector.

### 2.3. X-ray Phase Analysis

X-ray diffraction (XRD) measurements were carried out using the D8 Discover apparatus (Bruker, Billerica, MA, USA) equipped with the Cu K_α_, 0.15406 nm wavelength X-ray excitation source. The film samples were subjected to the measurements in the theta/2 theta geometry.

### 2.4. UV-Vis Test

The transmission spectra of the obtained samples were studied using the SF-56 spectrophotometer (LOMO, Saint Petersburg, Russia,) in the wavelength range 190–1100 nm.

### 2.5. X-ray Photoelectron Spectroscopy

The chemical composition of the surface of the obtained samples was analyzed by X-ray photoelectron spectroscopy (XPS). The XPS spectra were measured under ultra-high-vacuum (UHV) conditions of 10^−7^ Pa using the Escalab 250Xi X-ray photoelectron spectrometer (Thermo Fisher Scientific Inc., Waltham, MA, USA) with a photon energy of Al-Kα equal to 1486 eV. The XPS peak deconvolution was carried out by means of Shirley background subtraction followed by a peak fitting to Voigt functions with mixed Gaussian and Lorentzian character. The surfaces under study were sputtered using an Ar^+^ ion beam at 500 eV beam energy for 30 s. Such a surface cleaning method has proven its ability to reduce the relative concentration of carbon-containing and oxygen-containing contaminants down to 5–15% in organic and metal oxide materials [28,29]. The samples were stored at air atmosphere for 1 month after the synthesis until they were placed into UHV in order to carry out the XPS experiments.

### 2.6. Study of the Photocatalytic Properties

The photocatalytic properties of all the obtained samples were examined in the model chemical reaction of decomposition of brilliant green in the ultraviolet (UV) radiation range. For this purpose, a dye solution with 5 ppm concentration was prepared; the specific volume of the solution per unit area in the film under study was 20 mL/cm^2^. The area of the photocatalyst film was 18.75 cm^2^; the study was conducted at room temperature (25 °C). A linear 8 W UV lamp with emission maxima at 185 nm and 254 nm (WL 2001, lamp type T5 G5, Camelion, Shenzhen, China) was employed as a radiation source. The distance from the lamp to the solution surface was 2 cm, and the distance from the lamp to the sample surface was 5 cm. During the experiment, 3 mL test samples were extracted from the solution every 30 min, followed by spectrophotometric measurement of the absorption coefficient at the wavelength of 624 nm, corresponding to the maximum absorption of the brilliant green solution, and recalculation of its concentration according to the Bouguer–Lambert–Beer law [30]. Based on the data obtained, the constants for the rate of photocatalytic decomposition of the dye were calculated. To exclude decreases in the concentration of brilliant green in the solution for reasons not related to photocatalysis, such as adsorption of dye molecules on the film or direct decomposition in the solution under UV radiation, “blank” experiments were performed simultaneously. These data were taken into account in the final calculation of the concentrations.

## 3. Results and Discussion

### 3.1. Structure and Phase Composition

**Figure 1:**
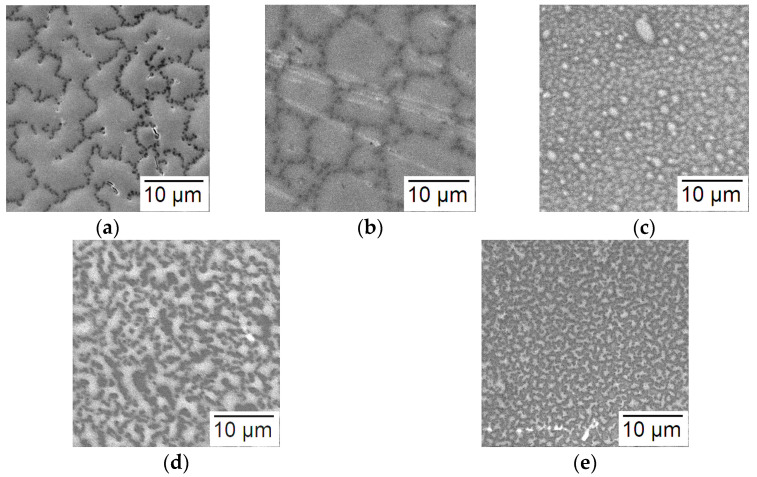
SEM images of the surface of samples under study: (**a**) PL0; (**b**) PL1; (**c**) PL2; (**d**) PL3; and (**e**) PL4.

Figure 1a shows the microstructure of the surface of the pristine sample PL0 obtained using scanning electron microscopy. It is clear that the sample has a labyrinth structure formed as a result of spinodal decomposition of the sol and consists of pre-percolation fragments whose growth occurs at different stages of maturation.

As a rule, the basic level in the hierarchy of the materials of this kind is represented by fractal aggregates similar in shape to spherical ones with sizes of 30–50 nm. A more detailed overview of the formation of these kinds of hierarchical structures in the sol-gel processes is given in a previous work [27].

The impact of plasma on the samples led to a noticeable rearranging the surface architecture (Figure 1b–e), which seems to be a consequence of the solid-phase recrystallization occurring in finely dispersed samples composed of crystallites smaller than 3 nm (as it will be shown below) at temperatures below the melting point. First of all, the “soft” treatment modes, at a source power of 250 W, led to the appearance of a granular structure with different sizes of agglomerates. When using the N_2_ plasma, the average size of the agglomerates was approx. 7–10 μm (Figure 1b), while O_2_ plasma treatment yielded agglomerates of about 1 μm size (Figure 1c). Applying the “harder” plasma modes resulted in fractal-labyrinth structures but with smaller characteristic dimensions than those of the PL0 sample (Figure 1d–e).

**Figure 2:**
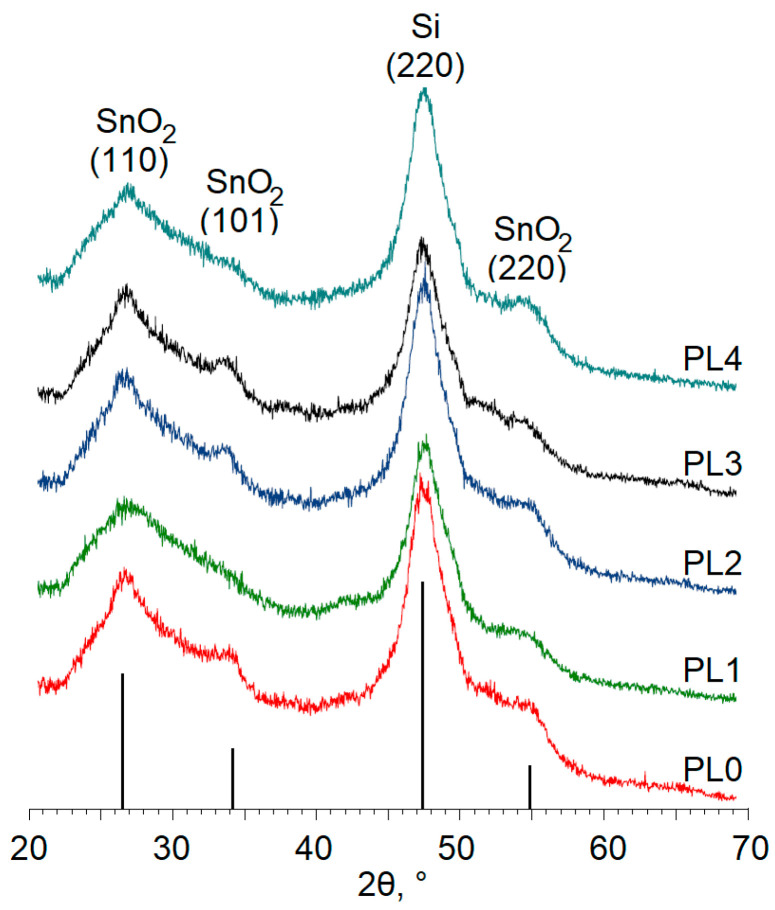
XRD patterns of the samples under study.

The results of the X-ray phase analysis presented in Figure 2 show that all types of the samples had reflections corresponding to the tin dioxide phase of the “cassiterite” (tin stone) type, which corresponds to the families of planes (110), (101) and (220). An intense reflection was also observed corresponding to the family of planes (220) of the Si substrate on which the films were formed. No other phases were found in the samples.

The analysis of the diffraction patterns indicates that the reflections corresponding to SnO_2_ were strongly broadened and characterized by low intensities. This finding may indicate, (1) the amorphousness of the samples, (2) the fine dispersion with the crystallite sizes less than 3 nm, and (3) the presence of micro stresses in the structure of the 0.85SnO_2_–0.15SiO_2_ film. However, considering the thermal annealing carried out for all the samples at a temperature of 600 °C and the depth of the plasma treatment, the second assumption is not unreasonable. Based on the Scherrer equation, the dimensions of the regions of coherent scattering *D* in the cassiterite phase are calculated for each sample as

$$D=\frac{K\lambda }{B_{hkl}\cos\theta },$$

where *K* = 0.9 is a numerical factor frequently referred to as the crystallite-shape factor, λ is the wavelength of the X-rays, *B_hkl_* is the width (full-width at half-maximum) of the X-ray diffraction peak in radians, and θ is the Bragg angle [31]). The calculation results are presented in Table 2.

These results reveal that all the modes of plasma treatment led to reductions in the sizes of coherent scattering regions. This effect was most pronounced for treatments of sample PL4: plasma processing forced the value D of the SnO_2_ phase to drop by almost a factor of 2, from 1.6 nm to 0.9 nm. It should be also noted that the SiO_2_ matrix present in the sample appeared in the X-ray amorphous state; as shown further by the XPS study, there is a presence of Si^4+^ ions in all the films.

**Figure 3:**
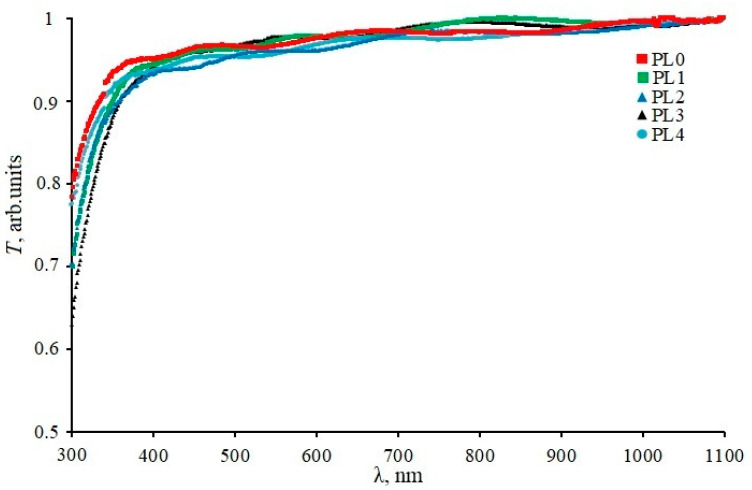
UV–Vis spectra of the samples under study.

Figure 3 shows the optical transmission spectra for all the obtained samples in the ultraviolet and visible radiation ranges.

We saw that for all the samples, the transmission of radiation in the entire studied range practically did not change, and in the wavelength range 400–110 nm it was about 0.95. The obtained spectra were used to calculate the values of the optical band gap of the samples. We found that this value practically did not change during film processing and amounted to 3.89 ± 0.04 eV.

### 3.2. XPS Results

**Figure 4:**
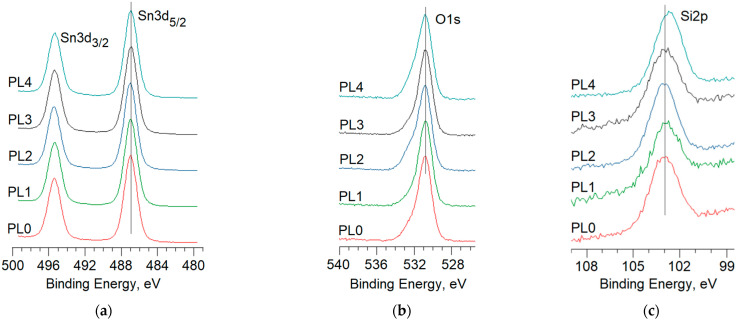
X-ray photoelectron spectra of the samples under study: (**a**) Sn 3d; (**b**) O 1s; and (**c**) Si 2p.

Figure 4 presents the spectra of Sn 3d, O 1s and Si 2p for each series of the produced samples. Preliminary etching of the thin near-surface layer of 0.85SnO_2_–0.15SiO_2_ with Ar ions in the spectrometer chamber to the depth of 10 nm made it possible to standardize the role of the adsorbates appearing on the surface due to storage in the air atmosphere. The energy positions of the spectra were calibrated by the binding energy of C 1s, whose value is equal to 285.0 eV and corresponds to the C–C bond of graphite [32].

**Figure 5:**
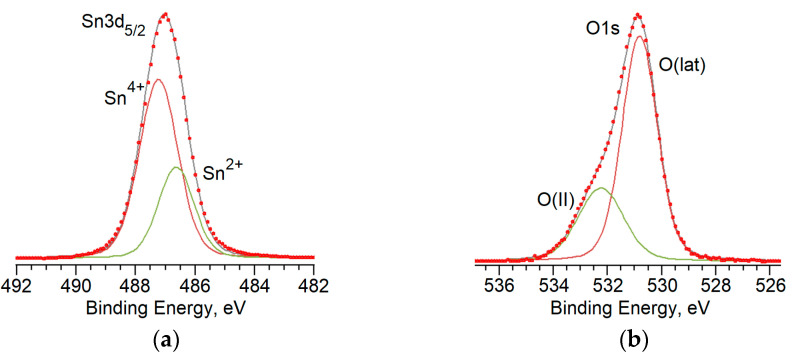
Deconvolution of the XPS spectra for Sn 3d_5/2_ (**a**) and O1s (**b**) energy regions for the PL2 sample.

The spectrum of Sn 3d given in Figure 4a is represented by a doublet with the binding energies of ~495.5 eV and ~487.0 eV corresponding to Sn 3d_3/2_ and Sn 3d_5/2_. The energy value indicates the mixed valence state of tin cations and the presence of Sn^4+^ and Sn^2+^ forms [33]. For example, Figure 5a gives the deconvolution of the Sn 3d_5/2_ spectrum in the PL2 sample into two components: a high energy one at ~487.3 eV corresponding to Sn^4+^ cations, and low energy one at ~486.6 eV corresponding to Sn^2+^.

**Figure 6:**
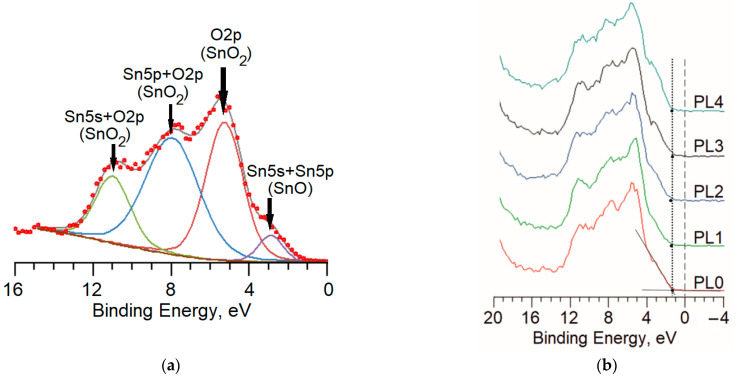
XPS of the valence bands for the samples under study: (**a**) example of the deconvolution for the PL0 sample; and (**b**) spectra of the PL0–PL4 samples.

It is worth noting that it is difficult to differentiate SnO and SnO_2_ oxides from the spectra of the core levels of tin due to the closeness of the binding energies of Sn^4+^ and Sn^2+^ [34]. In contrast, the spectrum of the valence band in the samples contains the features of both SnO and SnO_2_, which are easily distinguished from each other. This is clearly shown in Figure 6 which illustrates the deconvolution of the spectrum of the valence band for the sample PL0. Although interpreting the origin of the spectrum bands is a debatable issue, we have followed the approach described elsewhere [35]. The decomposition of the spectrum enables us to distinguish four components with the binding energies of 5.0, 8.0, 11.0 and 3.0 eV. The first band is associated with the states of O 2p, the second band links to the hybrid states of Sn 5s and O 2p, and the third band comes from the strong interaction between the orbitals of Sn 5s and O 2p. All the three components are attributed to Sn^4+^ cations in the tin dioxide phase. The component with the binding energy of 3.0 eV is a part of the SnO valence band and relates to the hybrid states of Sn 5s and Sn 5p [36].

The O 1s spectrum for all series of samples is asymmetric (Figure 4b) and it can be decomposed into two components (Figure 5b): O(lat) with the binding energy of ~531.0 eV, and O(II) with the binding energy of ~532.2 eV. The first low-energy component corresponds to oxygen surrounded by tin cations of Sn-O-Sn in the crystal lattice of SnO and SnO_2_ oxides [37]. The high-energy component of O(II) can link to oxygen anions in the crystal lattice of SiO_2_ [38], and the surface hydroxide groups adsorbed on the surface of the material, as well as the atomic and molecular forms of oxygen physically and chemically adsorbed on the surface of the tin dioxide [39]. A two-component O1s core level peak is commonly observed in many other metal oxides, such as ZnO [40]. Treatment of the sample PL2 with O_2_ plasma led to enhancement of the O(II) component, which is most likely to be the result of several factors. A more detailed differentiation of oxygen species on the surface of the material was carried out based on the quantitative data on the composition of the surface of the material. As given in Figure 4c, the spectrum of Si 2p for all series of samples is symmetrical; its maximum corresponds to the binding energy of 103.0 eV which corresponds to silicon in the oxidation state of Si^4+^ [41].

The ratios between the elements that make up the surface of the material were considered together with their various forms. All the parameters required for the analysis are listed in Table 3. The data on the chemical composition of the samples are presented without taking carbon into account and are reduced to 100% in total. The [Sn]/[Si] ratio in the pristine samples was 5.1, which is slightly higher than that in the initial sol where [Sn]/[Si] = 4.0. This indicates the formation of the composite of 0.83SnO_x_–0.17SiO_2_ and the coprecipitation of oxides close to the quantitative level (0.85SnO_2_–0.15SiO_2_ in the initial sol). The ratio of $\frac{\left[\mathrm{Sn}\right]}{\left[O(lat)\right]}$= 0.54 indicates a slight deviation from the stoichiometry of tin dioxide and the presence in the crystal structure of 15 at. % cations of Sn^2+^ in the total tin content, as well as the presence of oxygen vacancies which are the predominant type of the intrinsic electrically active point defects in SnO_2_ under normal conditions [42].

Assuming that the preliminary etching of the samples with Ar ions in the spectrometer chamber removed all surface adsorbates, the ratio of $\frac{2[\mathrm{Si}]}{\left[O(\mathrm{II})\right]}$ = 1.1 allows us to confirm that the state of the X-ray amorphous silicon dioxide is close to the stoichiometric state. It should be specifically noted that the possible reduction of SnO_2_ is unlikely to occur when using the indicated etching modes.

When considering the treatment of SnO_x_–SiO_2_ films with the inductively coupled N_2_ plasma source at the power of 250 W (sample PL1), the analysis of data in Table 3 indicates that this type of processing does not lead to the changes in the ratios of $\frac{\left[\mathrm{Sn}\right]}{\left[O(lat)\right]}$, $\frac{\left[Sn^{2+}\right]}{\left[\mathrm{Sn}\right]}$ or $\frac{2[\mathrm{Si}]}{\left[O(\mathrm{II})\right]}$. It proves that the values of the stoichiometry of the SnO_x_ and SiO_2_ oxides are preserved. In addition, increases in the ratio of $\frac{\left[\mathrm{Sn}\right]}{\left[\mathrm{Si}\right]}$ to the value of 8.0 indicates the predominant removal of silicon dioxide from the surface with almost unchanged SnO_x_.

Treatment of the films with inductively coupled O_2_ plasma at the power of 250 W (sample PL2) led to a significant change in the ratios between the chemical elements and their forms on the surface. First of all, we observed an enhanced ratio of $\frac{\left[\mathrm{Sn}\right]}{\left[O(lat)\right]}$ to the value of 0.62, which results from a higher contribution of Sn^2+^, up to 39% of the total amount of tin. These data suggest that lattice oxygen was removed and the surface of the material was enriched with oxygen vacancies. The possible oxidation of Sn^2+^ by atomic oxygen under the employed parameters of the plasma treatment seems to make a smaller contribution compared with removing the lattice oxygen. This is probably linked to the presence of reactive atomic oxygen, O*, in the plasma which reacts with oxygen in the crystal lattice to form the stable molecule of O_2_:

O* + O(*lat*) → O_2_(gas).

The absence of significant changes in the ratio of $\frac{2[\mathrm{Si}]}{\left[O(\mathrm{II})\right]}$ when compared with the pristine sample PL0 shows a weak interaction between the oxygen plasma and SiO_2_. In this case, the value of [Sn]/[Si] = 3.7 indicates the formation of the 0.79SnO_x_–0.21SiO_2_ composite, which slightly differs from the ratio of the components in the initial sol. This appears to be a consequence of partial surface etching when SnO_x_ is predominantly removed. This assumption corresponds to a change in the surface morphology of the samples (Figure 1c).

Increasing the power of the high-frequency source of inductively coupled N_2_ plasma up to 500 W (sample PL3) led to more noticeable changes in the surface of the studied material compared with the conditions at 250 W. Firstly, an increase in the ratio of $\frac{\left[\mathrm{Sn}\right]}{\left[O(lat)\right]}$ from 0.54 to 0.56 led to a higher contribution of the fraction of the Sn^2+^ form to the total content of Sn in the sample from 15% to 21%, which indicates the formation of oxygen vacancies on the surface in the sub-lattice of SnO_x_. Secondly, a significant increase of $\frac{2[\mathrm{Si}]}{\left[O(\mathrm{II})\right]}$ from 1.1 to 1.6, together with the practically unchanged ratio of $\frac{\left[\mathrm{Sn}\right]}{\left[\mathrm{Si}\right]}$, indicates a removal of oxygen from silicon dioxide. As a result, the remaining Si cations are not etched into the medium but rather transferred to the matrix of SnO_x_, replacing tin cations with generating Sn–O–Si bonds [33]. Presumably, the increased power of the plasma source allows transitions of this kind to occur. At the same time, it should be noted that due to the differences in the radii of Sn^4+^ and Si^4+^ cations, 71 pm and 42 pm, respectively, local mechanical stresses can arise in the material.

Connecting the second high-frequency source of inductively coupled N_2_ plasma with a power of 10 W to the main source with a power of 250 W has several effects on the surface of the SnO_x_-SiO_2_ films (sample PL4). Similar to the case of using a single source of 500 W, we observed an increase in the ratio of $\frac{\left[\mathrm{Sn}\right]}{\left[O(lat)\right]}$ from 0.54 to 0.56 in the samples, which also indicates the higher contribution of the Sn^2+^ form to the total content of Sn in the sample from 15% to 21%. In this case, a significant increase of $\frac{2[\mathrm{Si}]}{\left[O(\mathrm{II})\right]}$ up to 1.9 was observed with a simultaneous reduction in the binding energy of Si 2p from 103.01 eV down to 102.72 eV. This presumably indicates the discharge of oxygen from silicon dioxide and the formation of Si cations due to a lack of neighbors.

It should be also noted that in samples PL1–PL4, none of employed plasma treatments resulted in a change in the position of the Fermi level relative to the top of the valence band (Figure 6b), which is frequently called Fermi level pinning. This effect is considered in detail in the previous works [43,44].

### 3.3. Photocatalytic Properties

**Figure 7:**
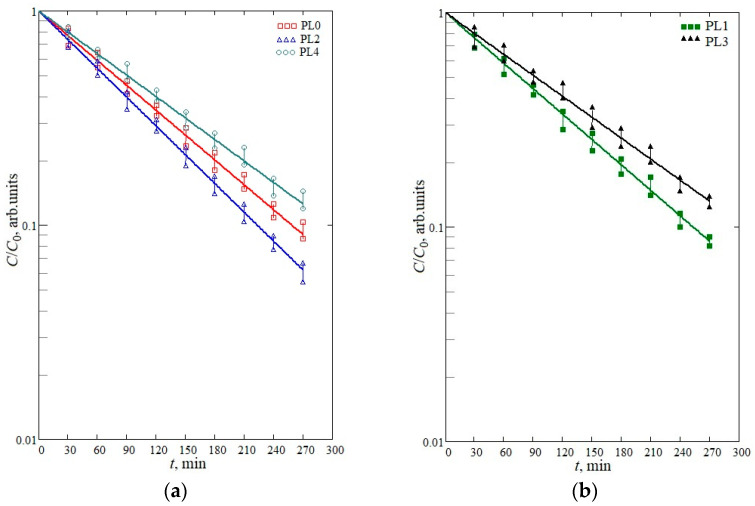
Photocatalytic decomposition curves for the PL0, PL2, PL4 (**a**) and PL1, PL3 (**b**) samples.

Figure 7 shows the kinetic curves of the photocatalytic decomposition of the dye on all the samples under study.

The experiments illustrate that the photocatalytic decomposition of the brilliant green solution in all the types of samples is a pseudo-first-order reaction, i.e., it obeys the kinetic equation:

$$c(\tau )=c_{0}\exp(-k\tau )$$

where *c(τ)* is the dye concentration at the time *τ*, $c_{0}$ is the initial concentration, and *k* is the reaction rate constant. The calculated values of the rate constants for each of the samples are given in Table 4. It should be noted that these values are relative in nature because they depend both on the volume of the solution to be directly in contact with the sample, and the area and thickness of the film; therefore, they reflect a qualitative change in the catalytic activity of the surface.

To analyze the obtained results, we may note the next major changes in the properties of films to appear as a result of plasma processing which contribute to the efficiency of the photocatalytic processes:

- A growing amorphization of the material. It is well known that increasing the degree of amorphism in the material adversely affects the photocatalytic properties of the material [45]. This is presumably linked to localized states appearing in the band gap which serve as centers for recombination of photogenerated charge carriers [46];
- The formation of vacancies in the oxygen sub-lattice at the near-surface layer. It was found in previous works [33] that their presence on the surface of tin dioxide improves the photocatalytic activity of the material. This type of vacancy is a trapping center for photogenerated holes, which prevents a recombination process. Also, oxygen vacancies are centers for capturing particles from the surrounding atmosphere, for example, dissolved molecular oxygen. This type of defect appears at maximum levels when the material is treated with O_2_ plasma. This is probably due to the interaction of the atomic charged form of oxygen with lattice oxygen, the generation of molecular oxygen and its desorption;
- The formation of heterojunctions of SnO_2_–SnO, where the photogenerated charge carriers are efficiently separated [47]. Thus, the resulting value of the rate constant for the reaction of photocatalytic oxidation is determined by the dominant factor in the combination of the factors.

In all cases, nitrogen plasma treatment either did not change the photocatalytic properties (PL1 sample) or worsened them (PL3, PL4 samples). This is presumably linked to the decisive contribution of the increasing amorphization in the material under its treatment and the small fractions of the Sn^2+^ and SnO forms. In contrast, the oxygen plasma mode (PL2 sample) was the only treatment that led to excitation of the photocatalytic activity. When using this mode, the proportion of the surface Sn^2+^ form reached ~40%, which indicates the appearance of the SnO_2_–SnO heterojunction and the high concentration of oxygen vacancies. Apparently, these two factors overcome the negative effect of the material amorphization. It should also be noted that generating oxygen vacancies, which is accompanied by the release of O^2−^ into the sublattice, leads to the formation of peroxide ions, which enhances photocatalysis. This fact was established by the DFT method and reflected in the work [48]. In this regard, we should expect an increase in photocatalytic activity with an increase in the density of vacancies in the oxygen sublattice.

## 4. Conclusions

This work explores the effect of O_2_ and N_2_ plasma treatment applied to thin semiconductor films based on tin dioxide derived by the sol-gel method. By means of the XPS and SEM methods, it was established that the pristine samples have the chemical composition of 0.83SnO_2_–0.17SiO_2_ under the developed labyrinth structure. We found that the phase composition of the initial samples is represented by cassiterite crystals with a size of ~1.6 nm. We found that all the types of plasma treatments led to increased dispersion of the material. “Soft” treatment with nitrogen plasma at a source power of 250 W did not lead to a noticeable change in the surface composition of the material. However, the photocatalytic activity of the samples, which was estimated using the model chemical reactions of brilliant green decomposition in the UV range, decreased. This was presumably due to decreasing crystallite size. Increasing the power of the inductively coupled plasma source up to 500 W led to the formation of oxygen vacancies on the surface and increased contribution of the Sn^2+^ form. Despite the fact that such point defects are trapping centers for photogenerated holes, as well as adsorption centers for oxygen dissolved in water, the photocatalytic activity of the samples also reduced. We suggest that it is linked to the decisive contribution of the increasing dispersion of the samples. Connecting the additional high-frequency plasma generator with a power of 10 W to the main one with a power of 250 W led to an effect similar to that observed when using nitrogen plasma with a source power of 500 W. This work demonstrated that O_2_ plasma treatment of pristine samples at 250 W power with a high-frequency source of inductively coupled plasma for 30 s led to an increase in the [Sn^2+^]/[Sn^4+^] ratio on the surface by a factor of 3.5, which was the maximum value among all the employed plasma modes. This presumably contributes to enhancing the photocatalytic activity by a factor of 1.15 by generating surface oxygen vacancies.

In general, we established that the resulting photocatalytic properties of the material are determined by the balance of three main parameters: the density of the surface oxygen vacancies; the degree of amorphization of the material; and the specific surface area of SnO_2_–SnO heterojunctions. All the plasma treatment modes led to an increase in the amorphization of the materials; however, O_2_ plasma resulted in the maximum density of oxygen vacancies on the surface. Specifically in this sample, we observed that the rate constant of the photocatalytic decomposition of brilliant green increased from 4.8 min^–1^ cm^–2^ to 5.5 min^–1^ cm^–2^. Treatment with N_2_ plasma led to a deterioration in the photocatalytic properties in all modes.

The results reported in this study demonstrate that oxygen plasma treatment of materials based on tin dioxide is a promising method for advancing their photocatalytic activity. Based on the observed effects, the plasma surface processing appears to be an effective technique with undiscovered and overlooked potential for producing device units based on SnO_2_–SiO_2_ nanocomposites for numerous sensor, adsorption and catalytic applications involving wide-gap semiconducting metal oxides.

## Figures and Tables

**Table 1:** Modes and parameters of plasma treatment of samples.

Treatment Parameter	Treatment Mode			
Power of high-frequency source of inductively coupled plasma, W	**PL1**	**PL2**	**PL3**	**PL4**
	250	250	500	250
Power of additional high-frequency source, W	0	0	0	10
Gas	N_2_	O_2_	N_2_	N_2_

**Table 2:** Dimensions of the regions of coherent scattering D in the samples under study.

Sample	PL0	PL1	PL2	PL3	PL4
D, nm	1.6	1.1	1.2	1.4	0.9

**Table 3:** Chemical composition and some ratios of elements and their forms for all series of samples under study.

Sample	(Sn), at. %	(Si), at. %	(O), at. %	$\frac{\left[\mathrm{Sn}\right]}{\left[\mathrm{Si}\right]}$	$\frac{\left[O(lat)\right]}{\left[O\right]}$	$\frac{\left[\mathrm{Sn}\right]}{\left[O(lat)\right]}$	$\frac{\left[Sn^{2+}\right]}{\left[\mathrm{Sn}\right]}$	$\frac{2[\mathrm{Si}]}{\left[O(\mathrm{II})\right]}$
PL0	29.3	5.8	64.9	5.1	0.84	0.54	0.15	1.1
PL1	31.3	3.9	64.8	8.0	0.89	0.54	0.15	1.1
PL2	28.0	7.6	64.4	3.7	0.69	0.62	0.39	0.8
PL3	31.6	5.5	62.9	5.7	0.89	0.56	0.21	1.6
PL4	28.7	9.7	61.6	3.0	0.83	0.56	0.21	1.9

**Table 4:** Rate constants for the decomposition of brilliant green.

**Sample**	**k, ×10^–4^ min^–1^ cm^–2^**
PL0	4.8 ± 0.05
PL1	4.8 ± 0.05
PL2	5.5 ± 0.05
PL3	4.0 ± 0.05
PL4	4.1 ± 0.05

## Data Availability

Data are contained within the article.
